# Supplementary material for: The vertical position of visual information conditions spatial memory performance in healthy aging
Source: Commun Psychol. 2023 Jul 25;1:2. doi: 10.1038/s44271-023-00002-3 (PMC11290605; doi:10.1038/s44271-023-00002-3)
Supplement: Supplementary file 2 — Supplementary Information [file 44271_2023_2_MOESM2_ESM.pdf]

Supplementary Information

The Vertical Position of Visual Information Conditions Spatial Memory Performance in Healthy Aging

Marion Durteste, Louise Van Poucke, Sonia Combariza, Bilel Benziane, José-Alain Sahel, Stephen Ramanoël* & Angelo Arleo*

**These authors jointly supervised this work*

Supplementary Methods

We conducted a complementary analysis in order to compare MPT parameter differences between young and older participants. In other words, we wished to evaluate how the difference between I_{down} and I_{up} as well as between S_{down} and S_{up} changed across age groups. For this purpose, we summed trial frequencies across young and older participants and fit the aggregate data into a single extended MPT model (Supplementary Figure 1). The latter comprised the three trees of the *Source-Item* model, corresponding to the three stimulus types, doubled for each age group. Accordingly, each parameter was added a label "Y" or "O" depending on whether it belonged to the young or older group, respectively. Order constraints were used on the parameters of the model to investigate how item position and age group influenced item and spatial memory. To disentangle the main effects of these two factors and their interaction, we implemented the order constraints using reparameterization[1–3]. We assumed that the probabilities associated with item memory and spatial memory were higher in young adults than in older adults. Building on these order constraints, we created four auxiliary parameters and modified the model equations accordingly. The α auxiliary parameters were created such that:

$$I_{down}\_O = I_{down}\_Y * \alpha I_{down}$$

$$I_{up}\_O = I_{up}\_Y * \alpha I_{up}$$

$$S_{down}\_O = S_{down}\_Y * \alpha S_{down}$$

$$S_{up}\_O = S_{up}\_Y * \alpha S_{up}$$

We first established a baseline model against which the order-constrained model would be compared. The baseline model had to include an equality constraint for the model to be identifiable. In accordance, parameter *g* was set to be equal across groups (g_Y = g_O). The two restricted models were then constructed; one in which the auxiliary parameters related to item memory were set to be equal ($\alpha I_{down} = \alpha I_{up}$), and the other in which the auxiliary parameters related to spatial memory were set to be equal ($\alpha S_{down} = \alpha S_{up}$). We then applied the ΔG^2 difference test for equality constraints in order to determine whether the interaction between object position and age influenced item or spatial memory. This extended analysis was conducted in multiTree[4], a software that allows for automatic reparameterization of MPT models. It is important to note that this approach to studying MPT parameter differences has been criticized. Indeed, goodness of fit measures tend to be disproportionately influenced by the group with higher aggregate numbers if sample sizes are unequal[5]. Moreover, participant heterogeneity is here not considered. We thus stress that the results are to be taken with caution.

Supplementary Note 1

The baseline MPT model fit the data well ($G^2(1) = 1.59$, $p = 0.21$). The ΔG^2 difference test revealed that the order-constrained model for item memory fit the data significantly worse than the baseline model ($\Delta G^2(1) = 10.94$, $p = 0.00094$). Regarding spatial memory, the ΔG^2 difference test also revealed that the restricted model fit the data significantly worse than the baseline model ($\Delta G^2(1) = 6.07$, $p = 0.014$). These results imply that the differences between I_{down} and I_{up} and between S_{down} and S_{up} are not equivalent between young and healthy older populations. According to these order-constrained models, object position and age interact to influence both item memory and spatial memory.

Supplementary Figures

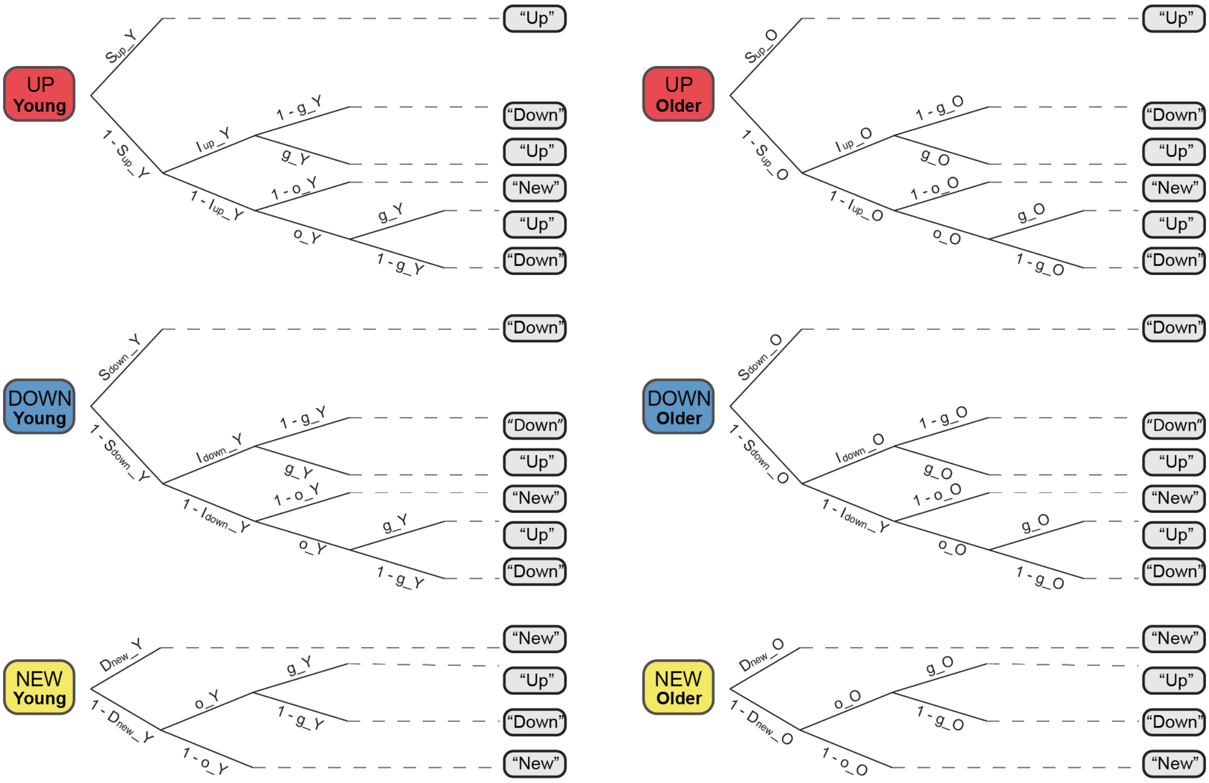

Supplementary Figure 1. **Graphical representation of the extended MPT *Source-Item* model used to analyze aggregated data from young and older participants**.

The *Source-Item* multinomial processing tree model from the adapted source monitoring paradigm, extended to young and older adults. Colored rounded rectangles represent the three different trial types: the object was presented in the upper part of the screen (UP), in the lower part of the screen (DOWN) or was not presented (NEW). Each tree is doubled as there is one stimulus type per age group in the extended MPT model. Grey rounded rectangles represent participants' possible answers. I_{up} and I_{down} are the probabilities of remembering an item that was presented in the upper visual field and lower visual field respectively. S_{up} and S_{down} are the probabilities of remembering the position of an item that was presented in the upper visual field and lower visual field respectively. Parameter o refers to the probability of guessing that an item was old while parameter g refers to the probability of guessing that an item was presented in the upper visual field. The Y and O labels indicate whether the parameter is being estimated from the young or older adult group, respectively.

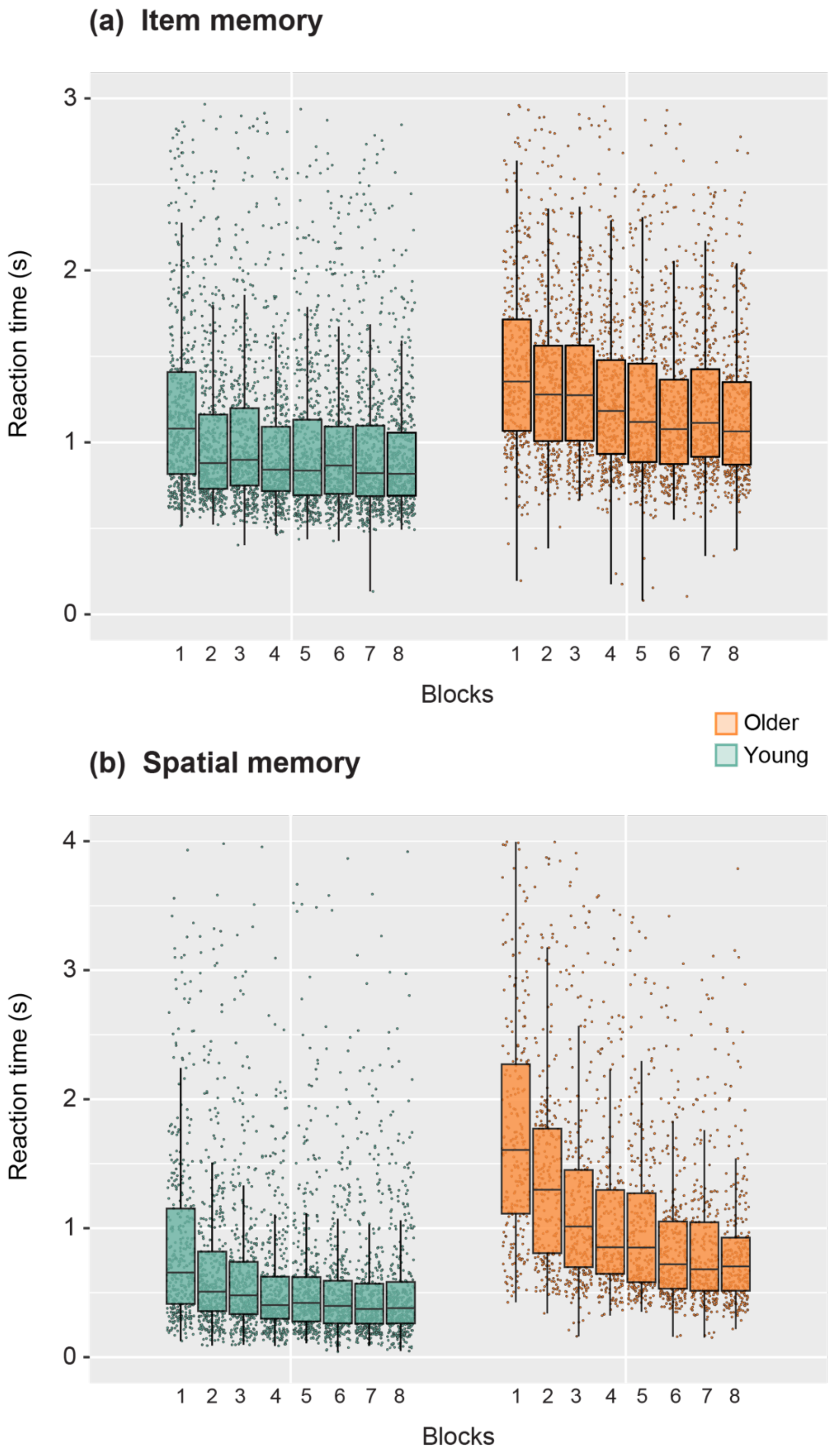

Supplementary Figure 2. **Reaction times in seconds across blocks and age groups.** Box plots depicting reaction times from the **(a)** item memory and **(b)** spatial memory tasks across the 8 experimental blocks (n = 25 young + 20 older participants). Participants improved their reaction times during the item memory ($\chi^2(7) = 343.77$, $p < 0.001$, $\eta^2 = 0.030$, 95% CI = [0.022, 0.040]) and during the spatial memory tasks ($\chi^2(7) = 867.37$, $p < 0.001$, $\eta^2 = 0.046$, 95% CI = [0.035, 0.056]). Each block is associated with a group-level whisker-and-box plot (center: median; box: 25th to 75th percentiles; whiskers: 1.5 × interquartile range). Each dot corresponds to a unique trial.

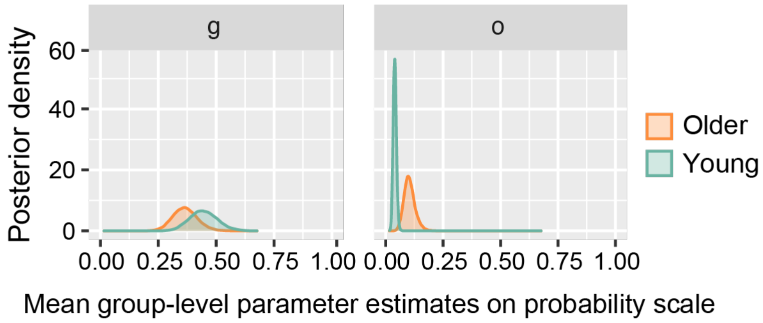

Supplementary Figure 3. **Guessing rates in young and older adults evaluated with MPT analyses**.
Posterior distributions depicting the results obtained using MPT analyses (n = 25 young + 20 older participants). The graphs show posterior distributions of the inverse-probit transformed group-level parameters related to guessing rates g and o on the probability scale. The parameter o describes the probability of guessing that the item was presented (i.e., answering that the item is old). The posterior distributions correspond to the updated knowledge about parameters g and o after considering the current data. The parameter g on the other hand describes the probability of guessing that the item was presented in the upper visual field.

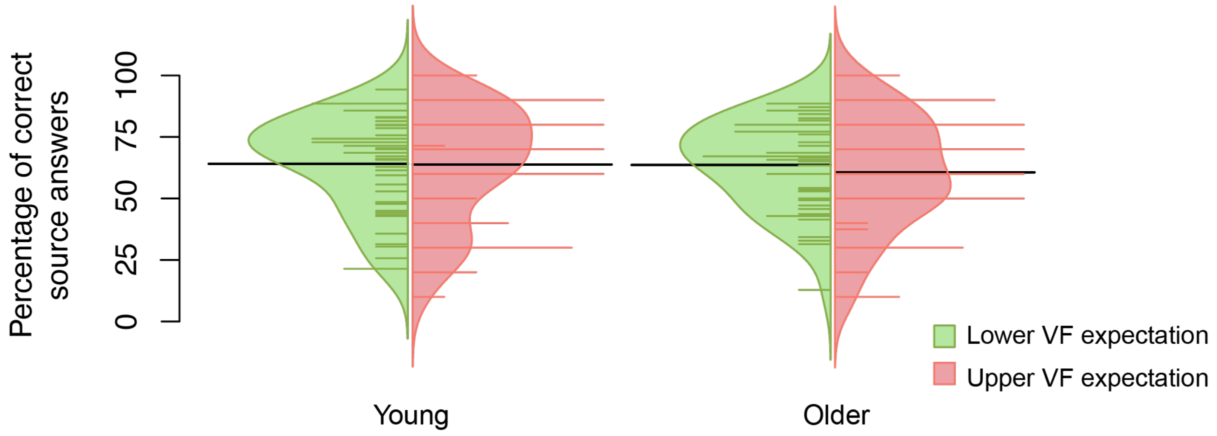

Supplementary Figure 4. **Spatial memory performance in young and older adults according to the source expectation of objects**.
Bean plots showing the percentage of correct answers during the spatial memory task in young and older adults (n = 25 young + 20 older participants). We found no evidence for a significant effect of pre-experimental source expectation on the percentage of correct answers during the spatial memory task ($F(1, 1164) = 1.20$, $p = 0.27$, $\eta_p^2 = 0.0010$, CI [0.00, 0.008]). Three independent raters assigned the position in space (i.e., upper or lower) that each item is most frequently associated with. The bean plots provide the density curve for each age group side-by-side, along with the individual data points displayed in a colored rug-plot. Bold horizontal black lines correspond to the mean of each group. VF = visual field.

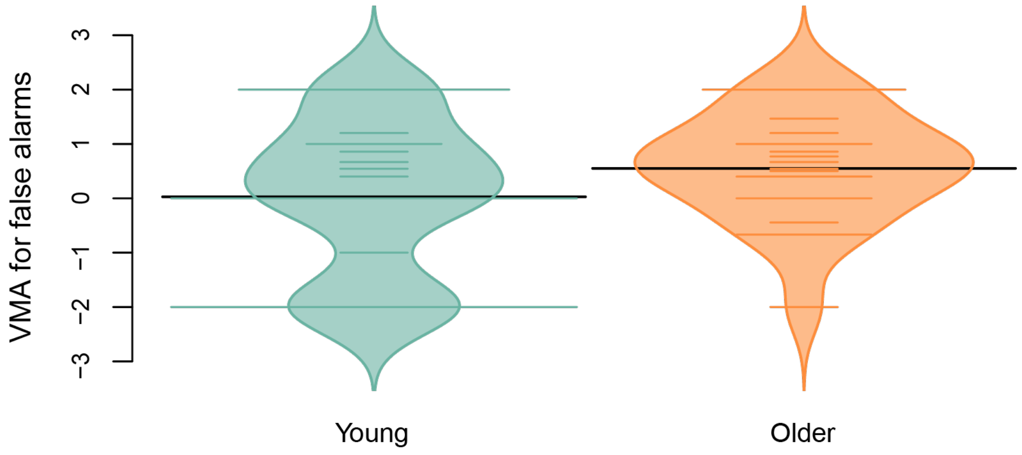

Supplementary Figure 5. **VMA for false alarms across age groups.**
Bean plots depicting the vertical meridian asymmetry (VMA) for false alarms in young and older adults (n = 25 young + 20 older participants). We found no evidence for a significant effect of age on the VMA for false alarms (R^2 = 0.021, F(1,42) = 1.92, *p* = 0.17, η_p^2 = 0.04, CI [0.00, 0.21]). A score of 0 indicates no difference between the proportion of upper and lower responses for false alarms. A score superior to 0 indicates a higher proportion of lower responses and a score inferior to 0 indicates a higher proportion of upper responses. The bean plots provide the density curve for each age group separately, along with the individual data points displayed in a colored rug-plot. Bold horizontal black lines correspond to the mean VMA of each group.

Supplementary Tables

	**Young adults** 11 M / 14 F	**Older adults** 8 M / 12 F
	Mean (± SD)	Mean (± SD)
**MMSE**	29.2 (± 1.1)	28.2 (± 1.5)
**3D Mental Rotation**	15.9 (± 5.7)	8.5 (± 5.3)
**Corsi Forward**	6.3 (± 1.1)	4.5 (± 0.8)
**Corsi Backward**	6.0 (± 1.2)	4.5 (± 0.8)
**Perspective-taking**	23.8 (± 19.1)	51.2 (± 28.2)

Supplementary Table 1. **Summary of young and older participants' scores on various neuropsychological tests**.
Scores are reported for the MMSE (mini mental state examination), the 3D mental rotation task, the Corsi forward and backward tasks and the perspective-taking test. M: male participants; F: female participants; SD: standard deviation.

Supplementary References

1. Knapp, B. R. & Batchelder, W. H. Representing parametric order constraints in multi-trial applications of multinomial processing tree models. *J. Math. Psychol.* **48**, 215–229 (2004) doi:10.1016/j.jmp.2004.03.002.
2. Kuhlmann, B. G., Erdfelder, E. & Moshagen, M. Testing Interactions in Multinomial Processing Tree Models. *Front. Psychol.* **10**, 2364 (2019) doi:10.3389/fpsyg.2019.02364.
3. Schmidt, O., Erdfelder, E. & Heck, D. W. Tutorial on multinomial processing tree modeling: how to develop, test, and extend MPT models. *PsyArXiv Preprint* (2022) doi:10.31234/osf.io/gh8md.
4. Moshagen, M. Multitree: A computer program for the analysis of multinomial processing tree models. *Behav. Res. Methods* **42**, 42–54 (2010) doi:10.3758/BRM.42.1.42.
5. Cooper, E., Greve, A. & Henson, R. N. Assumptions behind scoring source versus item memory: Effects of age, hippocampal lesions and mild memory problems. *Cortex* **91**, 297–315 (2017) doi:10.1016/j.cortex.2017.01.001.
