# Supplementary material for: The vertical position of visual information conditions spatial memory performance in healthy aging
Source: Commun Psychol. 2023 Jul 25;1:2. doi: 10.1038/s44271-023-00002-3 (PMC11290605; doi:10.1038/s44271-023-00002-3)
Supplement: Supplementary file 3 — Reporting Summary [file 44271_2023_2_MOESM3_ESM.pdf]

nature portfolio

Corresponding author(s): Angelo Arleo & Stephen Ramanoël

Last updated by author(s): Apr 26, 2023

# Reporting Summary

Nature Portfolio wishes to improve the reproducibility of the work that we publish. This form provides structure for consistency and transparency in reporting. For further information on Nature Portfolio policies, see our Editorial Policies and the Editorial Policy Checklist.

## Statistics

For all statistical analyses, confirm that the following items are present in the figure legend, table legend, main text, or Methods section.

| n/a | Confirmed | |
|---|---|---|
| ☐ | ☒ | The exact sample size (*n*) for each experimental group/condition, given as a discrete number and unit of measurement |
| ☐ | ☒ | A statement on whether measurements were taken from distinct samples or whether the same sample was measured repeatedly |
| ☐ | ☒ | The statistical test(s) used AND whether they are one- or two-sided<br>*Only common tests should be described solely by name; describe more complex techniques in the Methods section.* |
| ☐ | ☒ | A description of all covariates tested |
| ☐ | ☒ | A description of any assumptions or corrections, such as tests of normality and adjustment for multiple comparisons |
| ☐ | ☒ | A full description of the statistical parameters including central tendency (e.g. means) or other basic estimates (e.g. regression coefficient) AND variation (e.g. standard deviation) or associated estimates of uncertainty (e.g. confidence intervals) |
| ☐ | ☒ | For null hypothesis testing, the test statistic (e.g. *F*, *t*, *r*) with confidence intervals, effect sizes, degrees of freedom and *P* value noted<br>*Give P values as exact values whenever suitable.* |
| ☐ | ☒ | For Bayesian analysis, information on the choice of priors and Markov chain Monte Carlo settings |
| ☐ | ☒ | For hierarchical and complex designs, identification of the appropriate level for tests and full reporting of outcomes |
| ☐ | ☒ | Estimates of effect sizes (e.g. Cohen's *d*, Pearson's *r*), indicating how they were calculated |

*Our web collection on statistics for biologists contains articles on many of the points above.*

## Software and code

Policy information about availability of computer code

| | |
|---|---|
| Data collection | All behavioral data were collected using PsychoPy v2020.2.10. All gaze data from the EyeLink 1000 Tower Mount were recorded via the ioHub event monitoring framework implemented in PsychoPy. |
| Data analysis | The CSV file containing the behavioral data and HDF5 file containing the eye tracking data were first processed using custom Python code (Python version 3.8.1 in Spyder IDE version 4.1.5. All statistical analyses were subsequently performed using R version 4.0.3 in RStudio version 1.4.1103 (R Core Team, 2020; RStudio Team, 2021). Custom codes are available via the OSF repository at: https://osf.io/unby4/ |

For manuscripts utilizing custom algorithms or software that are central to the research but not yet described in published literature, software must be made available to editors and reviewers. We strongly encourage code deposition in a community repository (e.g. GitHub). See the Nature Portfolio guidelines for submitting code & software for further information.

## Data

Policy information about availability of data

All manuscripts must include a data availability statement. This statement should provide the following information, where applicable:

- Accession codes, unique identifiers, or web links for publicly available datasets
- A description of any restrictions on data availability
- For clinical datasets or third party data, please ensure that the statement adheres to our policy

The experimental data that support the findings of this study are available via the OSF repository at: https://osf.io/unby4/

## Human research participants

Policy information about studies involving human research participants and Sex and Gender in Research.

| | |
|---|---|
| Reporting on sex and gender | In the manuscript, we used the term sex. Participants self-reported their sex assigned at birth. Findings apply to both male and female sexes. During study design, we aimed to include equivalent numbers of male and female participants in the young and older adult groups. After excluding participants, we had slightly more female participants in both age groups. There were 14 female participants and 11 male participants in the young group along with 12 female participants and 8 male participants in the older group. For the hierarchical MPT analysis, we included sex as a covariate. Sex-based statistical analyses were not conducted on the grounds that we did not have any tangible scientific hypothesis. |
| Population characteristics | We included young (29.1 ± 4.2 years old) and older adults (75.5 ± 3.7 years old) . During cohort enrollment we ensured that participants did not have a history of psychiatric, neurological or sensory disorders. Moreover, all participants had normal or corrected-to-normal vision. |
| Recruitment | Participants were recruited from the SilverSight cohort at the Institute of Vision in Paris, France. Participation was voluntary and all enrolled adults received a 30€ compensation. We cannot exclude the possibility of a self-selection bias linked to non random differences that characterize individuals who choose to enrol in a cohort study. In particular, older adults who take part in research experiments are usually in very good mental and physical form. It is therefore important for future studies to replicate the present findings using a more diverse sample of participants. |
| Ethics oversight | The Ethical Committee "CPP Ile de France V" (ID_RCB 2015-A01094-45, CPP N: 16122) approved the experimental procedures. |

Note that full information on the approval of the study protocol must also be provided in the manuscript.

# Field-specific reporting

Please select the one below that is the best fit for your research. If you are not sure, read the appropriate sections before making your selection.

☒ Life sciences ☐ Behavioural & social sciences ☐ Ecological, evolutionary & environmental sciences

For a reference copy of the document with all sections, see nature.com/documents/nr-reporting-summary-flat.pdf

# Life sciences study design

All studies must disclose on these points even when the disclosure is negative.

| | |
|---|---|
| Sample size | A total of 45 participants (n = 25 young, n = 20 older) were included in the current study. No formal statistical analyses were conducted to determine the sample size a priori. We nonetheless made sure that our sample size was in line with that used in previous studies investigating vertical visual field asymmetries across age groups (Brennan et al., Can. J. Exp. Psychol., 2017; Feng et al., Eur. J. Ageing, 2017; Tsurumi et al., Dev. Sci., 2022). |
| Data exclusions | 26 young adults and 26 older adults took part in the study initially. We excluded one young adult as they were diagnosed with a neurological condition after their participation. We also excluded 6 older adults due to poor eye tracking performance. They failed to maintain appropriate central fixation throughout the encoding phases (see the Eye Tracking section for details). |
| Replication | No replication of this study was performed |
| Randomization | Participants were chosen randomly from the SilverSight cohort, with the constraint that they had to be between the ages of 18 and 35 or 60 and 82. The cohort comprises approximately 350 participants. |
| Blinding | We were not blinded to group allocation as the groups were defined according to age. |

# Reporting for specific materials, systems and methods

We require information from authors about some types of materials, experimental systems and methods used in many studies. Here, indicate whether each material, system or method listed is relevant to your study. If you are not sure if a list item applies to your research, read the appropriate section before selecting a response.

## Materials & experimental systems

| n/a | Involved in the study |
| --- | --- |
| ☒ | ☐ Antibodies |
| ☒ | ☐ Eukaryotic cell lines |
| ☒ | ☐ Palaeontology and archaeology |
| ☒ | ☐ Animals and other organisms |
| ☒ | ☐ Clinical data |
| ☒ | ☐ Dual use research of concern |

## Methods

| n/a | Involved in the study |
| --- | --- |
| ☒ | ☐ ChIP-seq |
| ☒ | ☐ Flow cytometry |
| ☒ | ☐ MRI-based neuroimaging |
